# P-274. An Assessment of the Agreement between Qualitative and Quantitative Measures of CD4 Counts Amongst Persons Screened for an Advanced HIV Clinical Trial in Nigeria

**DOI:** 10.1093/ofid/ofaf695.495

**Published:** 2026-01-11

**Authors:** Olukemi A Adekanmbi, Yahaya Mohammed, Iorhen Akase, Oche Agbaji, Simji Gomerep, Uche Unigwe, Juliet Mmerem, Asukwo Onukak, Vivian Kwaghe, Garba Iliyasu, Chiedozie Maduka, Bassey Ekeng, Peter Nwakile, Dimie Ogoina

**Affiliations:** University College Hospital, Ibadan, Ibadan, Oyo, Nigeria; Usman Danfodiyo University Teaching Hospital, Sokoto, Sokoto, Nigeria; Lagos University Teaching Hospital, Lagos, Lagos, Nigeria; Jos University Teaching Hospital, Jos, Plateau, Nigeria; Jos University Teaching Hospital, Jos, Plateau, Nigeria; University of Nigeria Teaching Hospital, Nsukka, Enugu, Nigeria; University of Nigeria Teaching Hospital, Nsukka, Enugu, Nigeria; University of Uyo Teaching Hospital, Uyo, Akwa Ibom, Nigeria; University of Abuja Teaching Hospital, Abuja, Federal Capital Territory, Nigeria; Aminu Kano Teaching Hospital, Kano, Kano, Nigeria; Federal Medical Center Umuahia, Umuahia, Abia, Nigeria; University of Calabar Teaching Hospital, Calabar, Cross River, Nigeria; Niger Delta University Teaching Hopsital, Yenagoa, Bayelsa, Nigeria; Niger Delta University

## Abstract

**Background:**

Advanced HIV disease (AHD) is of great concern in developing countries like Nigeria. Nigeria implemented an AHD package of care that includes rapid qualitative point-of-care (POC) CD4 testing to identify individuals with CD4 counts ≤200 cells/mm^3^, guiding further screening (e.g. TB lipoarabinomannan and cryptococcal antigen tests). However, the accuracy of this qualitative method compared to gold-standard quantitative CD4 testing via flow cytometry remains uncertain. We aimed to evaluate the agreement between POC qualitative CD4 testing and gold standard for detecting CD4 counts ≤200 cells/mm^3^

**Methods:**

Data were collected retrospectively from records of individuals who had been screened for an AHD clinical trial from February to November 2024 at antiretroviral therapy (ART) clinics of 7 tertiary hospitals across 5 geo-political zones in Nigeria. Persons newly found to have acquired HIV or had interrupted treatment for at least 6 months were screened with a rapid POC qualitative CD4 assay as standard of care. Those with CD4 count ≤200 cells/mm3 according to the POC assay subsequently had quantitative CD4 testing as screening for the trial.

**Results:**

A total of 430 participants with a mean age of 40.8 years (±13.9 SD) were enrolled. A slight majority were females, 226(52.6%), and the Lagos University Teaching Hospital had the most participants [206(47.9%)]. Thirteen participants also had tuberculosis, while 1 each was found to have Hepatitis B and cryptococcal meningitis respectively. The qualitative POC CD4 method correctly identified 336(78.1%) out of 430 participants as having CD4 ≤200 cells/mm3 while misclassifying 94 out of 430 participants that actually had CD4 >200 cells/mm3 based on the gold standard.

**Conclusion:**

The qualitative POC CD4 method provides a rapid and cost-effective alternative to the gold standard but shows limitations, particularly near critical thresholds (≤200 cells/mm3). These limitations may impact clinical decisions and outcomes, suggesting the need for confirmatory quantitative testing where feasible. Rapid qualitative methods should complement, not replace, quantitative CD4 testing, especially in resource-limited settings where accurate risk stratification for treatment decisions are critical.

**Disclosures:**

All Authors: No reported disclosures

